# Start-Up of Chitosan-Assisted Anaerobic Sludge Bed Reactors Treating Light Oxygenated Solvents under Intermittent Operation

**DOI:** 10.3390/ijerph18094986

**Published:** 2021-05-07

**Authors:** Keisy Torres, Francisco Javier Álvarez-Hornos, Carmen Gabaldón, Paula Marzal

**Affiliations:** Research Group GI2AM, Department of Chemical Engineering, Universitat de València, 46100 Burjassot, Spain; kvtorresr@unal.edu.co (K.T.); carmen.gabaldon@uv.es (C.G.); Paula.Marzal@uv.es (P.M.)

**Keywords:** anaerobic reactors, chitosan, granulation, intermittent feeding, solvents, high-throughput sequencing

## Abstract

Quality of the granular sludge developed during the start-up of anaerobic up-flow sludge bed reactors is of crucial importance to ensure the process feasibility of treating industrial wastewater such as those containing solvents. In this study, the microbial granule formation from suspended-growth biomass was investigated in two chitosan-assisted reactors. These reactors operated mimicking industrial sites working with night closures treating a mixture of ethanol, ethyl acetate, and 1-ethoxy-2-propanol. Each reactor operated under different hydrodynamic regimes typical from UASB (R1: <0.15 m h^−1^) and EGSB (R2: 3 m h^−1^). High soluble COD removal efficiencies (>90%) accompanied by rapid formation of robust anaerobic granules were achieved at both up-flow velocity levels. After three weeks from the start-up, mean size diameters of 475 µm and 354 µm were achieved for R1 and R2, respectively. The performance of the process was found to be stable for the whole operational period of 106 days treating intermittent OLR up to 13 kg COD m^−3^ d^−1^. A memory dose of chitosan at day 42 was beneficial to guarantee good quality of the granules by offsetting the negative impact of intermittent water supply on the granular size. *Methanocorpusculum* was identified as the dominant archaea at both up-flow velocities. *Acetobacterium*, *Geobacter* and *Desulfovibrio* bacteria were also abundant, demonstrating its role on the degradation of light-oxygenated solvents.

## 1. Introduction

The circular economy has become the focus of social, economic, and environmental policies toward sustainable development. In 2020, the European Commission launched its second Circular Economy Action Plan, *A new Circular Economy Action Plan—For a cleaner and more competitive Europe* [1], reinforcing the strategy adopted in 2015. Special attention in this action plan is paid to packaging and plastics. The printed flexible packaging industry provides highly versatile products in various formats and materials all over the world. Mainly, they are supplied to sectors such as the food industry, but they are also used for pharmacy and medical products, cosmetics and toiletries, household products, and retail non-food products, among others. This is a growing sector that had a value output in Europe of €39.2 billion in 2018 [2]. The contribution of the flexographic sector to the circular economy and its principles focuses not only on product recyclability but also on closing the loop in the industrial production process by transforming waste gas emissions into bioenergy [3]. Such facilities consume high quantities of organic solvents causing emissions of volatile organic compounds (VOC), mainly from evaporation during the ink drying. A novelty approach for VOC control is the anaerobic bioscrubber, in which biological processes and physical unit operations are combined to recycle low-concentration VOC emissions into bioenergy [4]. The core of the anaerobic bioscrubber is based on the anaerobic degradation of light-oxygenated solvents in a high-rate expanded granular sludge bed reactor (EGSB). Excellent performance has been demonstrated in an industrial prototype inoculated with granular sludge from a brewery wastewater treatment plant. Solvent removal efficiencies > 93% were reported in the treatment of mixtures of ethanol, ethyl acetate, and 1-ethoxy-2-propanol [5]. The great potential of this technology for solvent recycling was demonstrated with the production of a biogas enriched in methane (> 94% CH_4_ content) with optimal yields (0.32 Nm^3^ CH_4_ kg^−1^ COD removed).

High-rate granular sludge bed systems are broadly applied during the treatment of industrial wastewater from not only the agro-food, beverage, alcohol distillery, or pulp and paper sectors but also in the chemical or pharmaceutical industries [6]. Applications to the treatment of wastewaters polluted with organic solvents have also been reported [7,8,9]. However, solvent-based wastewater can negatively impact the start-up and can even adversely affect the quality of the granules of up-flow anaerobic sludge blanket (UASB) or EGSB reactors, which in turn can impact the organic loading rate (OLR) that can be treated. This negative effect can be intensified under fluctuating wastewater flows associated with different work shifts in industrial manufacturing. For instance, Lafita et al. [10] reported partial disintegration of granules in an EGSB operated under intermittent wastewater supply with nightly shutdowns.

The formation and stability of granules is a key factor for the success of the operation and the mechanism of the sludge granulation process has been widely studied and elucidated at least for practical applications [6]. However, long start-up periods needed for a satisfactory development of the granules represent a major drawback for the industrial application of the process. In most cases, the strategy for a rapid start-up of UASB reactors has been inoculating them with granular sludge collected from other reactors in operation. The same strategy is implemented for seeding the EGSB reactors. Nevertheless, availability of granules can be limited and the cost of acquiring and transporting the granules can be substantial [11], highlighting the interest in methodologies for rapid development of stable granules from affordable and widely available flocculent sludge. For this purpose, the addition of external additives, such as ionic polymers, has been demonstrated to enhance granulation, thus accelerating the start-up [12,13,14]. Among ionic polymers, chitosan has shown positive results on sludge granulation along with advantages such as availability, environmental friendliness, and biodegradability [15]. Tiwari et al. [16] observed larger granules in chitosan-assisted UASB reactors continuously fed with sucrose low-strength wastewater than in the control reactor or those with *Reetha* extracts as additives. Hudayah et al. [17] reported a granule percentage of 52.8% with an average diameter of 535 μm at the end of the operation of a chitosan-added reactor fed semi-continuously with synthetic wastewater containing glucose and propionate. Along with better physicochemical characteristics, granules > 1000 μm appeared earlier and with a higher percentage in the chitosan-added reactor. Recently, chitosan has been also tested as a coadjuvant in bio-carriers such as polyvinyl alcohol gel beads [18] or biochar [19] to improve granulation. However, the application of chitosan for granulation in the presence of organic solvents is still scarce. In our previous work [20], successful development of granular sludge in continuously fed UASB reactors was achieved. The beneficial effects of single doses of chitosan were demonstrated in the treatment of wastewater containing light-oxygenated solvents. The shift of the operation to intermittent feeding, simulating industrial emission patterns, was detrimental to the granules’ size. In such cases, periodic addition of chitosan restored the stability in the size of the granules [21]. Nevertheless, the study of the granulation of flocculent sludge has not been reported by using experimental conditions that mimic industrial production (intermittent).

The present research aims to evaluate the chitosan-assisted formation and the evolution of anaerobic granules developed from flocculent sludge in anaerobic reactors treating wastewater polluted with a mixture of oxygenated organic solvents (1) under intermittent feeding and (2) under hydraulic regimes within those of UASB and EGSB reactor configurations. For this purpose, two reactors were operated for over 100 days at increasing OLR levels with an intermittent pattern of 16 h per day, 7 days per week. Performance of the operation and the evolution of the granules’ size were examined. The dynamics of the microbial communities were evaluated throughout the experiment to gain insight into the microbiology of the process.

## 2. Materials and Methods

### 2.1. Experimental Setup

**Figure 1:**
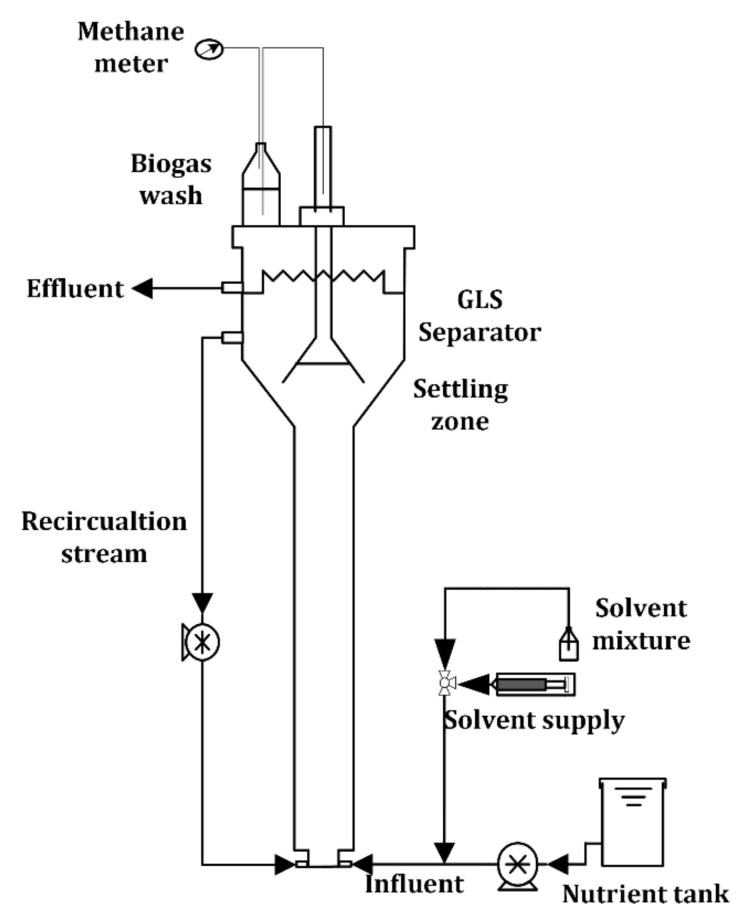
Scheme of the experimental setup.

Experiments were carried out in two PVC laboratory-scale reactors (R1 and R2) with an effective volume of 7.8 L (Figure 1). The reactors consisted of the reaction zone (internal diameter of 6.5 cm, height of 120 cm) plus the settling zone containing the gas-liquid-solid (GLS) separator (diameter of 20 cm, height of 24 cm). The reactors were fed with synthetic wastewater using a syringe pump (New Era, 1000 model, USA). The up-flow velocity was adjusted, if required, by effluent recirculation using a peristaltic pump (Watson-Marlow, USA). Both water flows were shared before entering the reactor. Biogas flowrate was measured after CO_2_ absorption with an NaOH solution.

### 2.2. Inoculum and Feed Characteristics

Each reactor was seeded with sludge from the anaerobic digester of the Quart-Benager municipal wastewater treatment plant (WWTP) located in Valencia (Spain). The anaerobic sludge had a total suspended solids (TSS) concentration of 17.8 g L^−1^ with a volatile suspended solids (VSS) content of 56 %, and a mean particle size of 85.3 μm. For the mixture of solvents tested in this study, the specific methanogenic activity (SMA) of the inoculum was 48 ± 1 NmL CH_4_ g VSS^−1^ d^−1^. The flocculation of the inoculated suspended-growth sludge was promoted by using chitosan (commercial-grade powder medium MW with a deacetylation grade of 75%, Sigma-Aldrich, Spain). The optimum chitosan concentration was determined by a jar test study with doses ranging from 2 to 24 mg chitosan g VSS^−1^. The inoculation procedure was done at day 0 as follows: 2.5 L and 1.8 L of inoculum were added to R1 and R2, respectively, together with a selected chitosan dose of 8.1 mg g VSS^−1^. A smaller quantity of inoculum was used in R2 to avoid washout due to the higher hydraulic load selected.

In order to emulate typical effluent composition from printing industries [5], a 7:2:1 mass ratio mixture of ethanol, ethyl acetate, and 1-ethoxy-2-propanol (E2P) was used for the duration of the study. The influent wastewater was buffered with NaHCO_3_ so as to keep the reactor pH at 7–7.5, and supplemented with macronutrients (COD:N:P ratio of 300:2:1) and micronutrients (Fe, B, Zn, Cu, Mn, Mo, Al, Co, Ni, EDTA) dosing as described in [20]. Additionally, calcium and magnesium were added to guarantee effluent concentrations > 150 mg Ca^+2^ L^−1^ and > 40 mg Mg^+2^ L^−1^.

### 2.3. Experimental Plan

Each reactor was operated under a different set point of the up-flow velocity to assess the influence of the hydraulic load on the biomass granulation. R1 was operated with increasing up-flow velocities from 0.05 to 0.15 m h^−1^, typical of UASB reactors. R2 was operated at a higher constant up-flow velocity of 3 m h^−1^, which is in the low range of up-flow velocities in EGSB reactors.

After inoculation, both reactors started simultaneously under intermittent feeding at room temperature (23.3 ± 1.8 °C). Synthetic solvent-based wastewater was supplied to R1 and R2 for 16 h per day, 7 days per week (intermittent loading mode with night closures). This feeding pattern was selected in order to evaluate the feasibility of biomass granulation under substrate supply conditions usually found at industrial sites. Intermittent feeding was maintained for 97 days and afterward the reactors were operated under continuous organic loading to assess the process performance in comparison with the intermittent mode. The experimental plan was conducted in four stages as indicated in (Table 1). The intermittent feeding period was divided into three stages (Stage I to III) with stepwise increments of organic loading rate (OLR) from < 2 (Stage I) to 13 kg COD m^−3^ d^−1^ (Stage III). To enhance the granulation process, three chitosan doses (8.1 mg g VSS^−1^) were added during the start-up on a weekly basis (on days 7, 14, and 21, Stage I). A memory chitosan dose was applied at the beginning of Stage II on day 42 to reinforce the granules’ stability. Finally, both reactors were operated under continuous organic loading (Stage IV). ORL was initially maintained with the maximum value applied in the intermittent wastewater supply period (13 kg COD m^−3^ d^−1^) and adjusted when necessary in accordance with the performance of the reactors.

### 2.4. Biochemical Methane Potential (BMP) and Specific Methanogenic Activity (SMA) Assays

Biomass samples were taken from both reactors at the end of the study (day 106) to determine the biodegradability of each solvent and its mixture with the granular sludge. The assays were carried out in triplicate at 25 °C using an automatic methane potential test system (AMPTS II, Bioprocess Control, Lund, Sweden). Bottles were filled with biomass and a nutrient medium at a ratio of 2.1 g VSS g COD^−1^. The nutrient medium, containing 2.5 g COD L^−1^ of each solvent (ethanol, ethyl acetate, and E2P) or the ternary mixture, was buffered with NaHCO_3_ and fortified with macro- and micronutrients at same concentration as the influent wastewater. The biochemical methane potential (BMP) was determined as the final accumulated methane production per initial organic carbon concentration and the specific methanogenic activity (SMA) was calculated at the maximum specific methane production rate. Methane recovery ranged between 89 ± 4% and the removal efficiency of organic carbon was > 95% for all the tests.

### 2.5. Particle Size Distribution, Settling Velocity and Strength of Granules

Biomass samples were taken from both reactors every 3 weeks and filtered through a 2 mm sieve to measure the quantity of particles with a diameter > 2 mm. The < 2 mm fraction was analyzed via laser diffraction to determine the particle size distribution (by volume) using a Mastersizer 2000 (Malvern Instruments Ltd.,Malvern, UK). Measures were done in triplicate. At the end of the study (day 106), settling velocity, strength, and morphology of the granules were determined. The settling velocity and the strength of the granules were determined according to the methods described by Ghangrekar et al. [22]. The settling velocity was estimated by measuring the average time taken for a single granule to settle at a certain height in a PVC column (diameter of 6 cm, height of 60 cm) filled with tap water. The strength of the granules was measured according to the integrity coefficient (IC) percentage range, which is defined as the ratio of solids in the supernatant to the total weight of granular sludge after a period of agitation. The granules were fixed, dried to the critical point, coated with Au-Pd, and then the morphology of the granules was examined using scanning electron microscopy (SEM-4100 model, Hitachi, Tokyo, Japan).

### 2.6. Analytical Methods

Effluent samples for each reactor were analyzed twice per week. Soluble COD, TSS, and VSS were determined according to the Standard Methods for the Examination of Water and Wastewater [23]. The effluent concentration of short-chain volatile fatty acids (VFA), expressed as acetic acid (mg HAc L^−1^), were measured from centrifuged samples using a titrator (848 Titrino Plus, Metrohm, Herisan, Switzerland). The solvent concentration was measured in a gas chromatograph (Agilent GC 7890A, Las Rozas, Spain) equipped with a Restek Rtx-VMS column (30 m × 0.25 mm × 1.4 mm) and a flame ionization detector and operated with 1.3 mL min^−1^ of helium as a carrier. The injector and detector temperatures were set to 190 °C and 240 °C, respectively. The oven temperature was set to 60 °C for 14 min followed by a ramp up of 25 °C min^−1^ to 110 °C. Biogas composition was analyzed in a gas chromatograph (Agilent GC 7820A, Las Rozas, Spain) equipped with a thermal conductivity detector and two columns connected in series, HP-Plot/U (30 m × 0.32 mm × 10 mm) and HPMolisieve (30 m × 0.32 mm × 12 mm), and operated with 3.5 mL min^−1^ of helium. The injector, oven, and detector temperatures were 200 °C, 40 °C, and 250 °C, respectively. Methane production was monitored by the volumetric gas meter of the AMPTS II (Bioprocess Control, Lund, Sweden)).

### 2.7. Microbial Community Analysis

Microbial analysis was performed on the inoculum and on the samples of sludge taken from both reactors on the last day (day 106) of the study. DNA was extracted from 0.5 g of sludge using the Power Soil DNA Isolation Kit (MOBIO Laboratories, USA). After the DNA was extracted, it was stored at −20 °C. The V4 hypervariable region was amplified with the universal primers 515F (5′-GTG CCA GCMGCC GCG GTA A-3′) and 806R (5′-GGACTA CHV GGGTWT CTA AT-3′). High-throughput sequencing was performed using a MiSeq System (Illumina, San Diego, USA). The raw 16S rRNA gene sequences were screened and trimmed by using the Quantitative Insights Into Microbial Ecology (QIIME) software [24] with a sequence length of 200 nt and mean quality score cutoff of 25 nt.

## 3. Results and Discussion

### 3.1. Performance of the Anaerobic Sludge Bed Reactors

**Figure 2:**
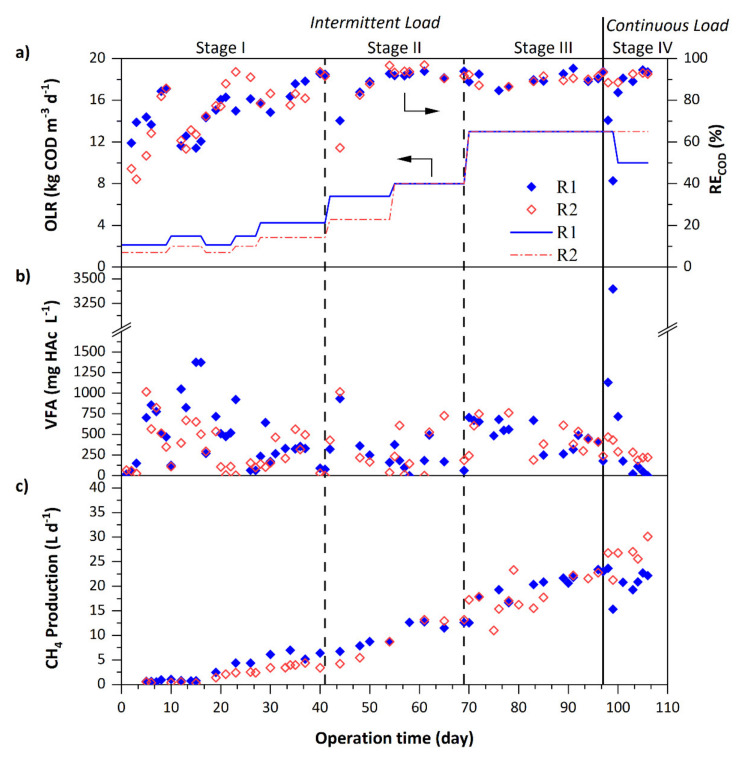
Performance of the anaerobic sludge bed reactors. (**a**) Organic load applied (―) and soluble COD removal efficiency (♦, **◊**), (**b**) VFA concentration in the effluent of the reactors (♦, **◊**), (**c**) methane production (♦, **◊**). R1 operated at U_L_ ≤ 0.15 m h^−1^ and R2 operated at U_L_ = 3.0 m h^−1^.

Figure 2 depicts the soluble COD removal efficiency (RE_COD_) along with the OLR applied, the VFA concentration in the effluent, and the methane production of both reactors (R1, R2) for the duration of the study. Data of intermittent stages (Stages I–III) correspond to measurements taken 8 h after the resumption of intermittent feeding. Stage I corresponds to the smooth start-up of the reactors while promoting the sludge granulation. During the first 9 days of operation at low OLR (< 2.5 kg COD m^−3^ d^−1^), RE_COD_ consistently increased up to 85% in both reactors (Figure 2a). After an early attempt to increase OLR (day 10), which caused a deterioration of the COD removal in both reactors, good removals (> 82%) were obtained from day 22. Therefore, OLR was progressively increased from then onward. At the end of the start-up (day 41, Stage I), both reactors maintained RE_COD_ > 90%. Nevertheless, the reactor operating at a lower up-flow velocity could treat greater OLR (4.3 kg COD m^−3^ d^−1^ in R1 versus 2.9 kg COD m^−3^ d^−1^ in R2). During the first four weeks of operation, VFA peaks (> 600 mg HAc L^−1^, Figure 2b) were measured due to the imbalance between the production and the degradation of volatile fatty acids. This indicated the lower activity of methanogenic microorganisms during the early working period. At the end of the start-up, VFA concentrations were < 100 mg HAc L^−1^, thus demonstrating that a good start-up and process stability can be achieved with intermittent feeding.

After the start-up, the increase in organic load on day 42 (Stage II) resulted in RE_COD_ decreases (70% in R1, 57% in R2) and VFA concentration increases (~1000 mg HAc L^−1^), but this transitory deterioration was recovered in a few days. Despite the increase in OLR up to 8 kg COD m^−3^ d^−1^, the RE_COD_ maintained > 90% in both reactors until the end of Stage II. Similar RE_COD_ values were reported by Bravo et al. [5] in a pilot EGSB reactor inoculated with granular sludge and fed with wastewater mainly contaminated with ethanol, ethyl acetate, and E2P. Data in Figure 2 show that in a period of less than 2 months it was possible to achieve high RE_COD_ using sludge in suspension as an inoculum combined with chitosan dosing. This can be considered a promising result for the reduction of the cost associated with the acquisition of granular sludge for industrial-scale facilities. Evolution of TSS in the effluent of both reactors (values ranging 30–120 mg L^−1^ from day 30 onward, data not shown) confirmed that despite the higher up-flow velocity of the liquid in R2 the loss of solids was low and comparable with that of R1. The low TSS in the effluents indicated the augment in solids retention time, which is related to the formation of granular biomass [25]. The next increase in OLR to 13 kg COD m^−3^ d^−1^ (day 70, Stage III) demonstrated the good adaptability of the microorganisms to periods without organic substrate supply. The adequate recovery of the microbial activity every day after feeding resumption enabled RE_COD_ > 90% with a VFA concentration < 500 mg HAc L^−1^ at the end of Stage III. The higher up-flow velocity in R2 did not have a significant effect on the system’s performance as reported by Jeison and Chamy [26] when comparing UASB vs EGSB reactors during continuous treatment of a stream contaminated with 10 g COD L^−1^ ethanol. During continuous supply of solvents (Stage IV), only R2 was able to process an OLR of 13 kg COD m^−3^ d^−1^ at high removals (> 93%). By contrast, R1 could treated lower OLR (10 kg COD m^−3^ d^−1^) to guarantee RE_COD_ > 90%. The better performance of R2 may be related to the improved contact between the biomass and the wastewater by working at high up-flow velocity.

Methane production (Figure 2c) began after the first 2 weeks and increased according to OLR increases. At the end of the intermittent feeding (day 97, Stage III), methane production was very similar in both reactors (~23.0 L d^−1^) while during continuous feeding greater values were obtained in R2 (27.0 L d^−1^) as higher organic loads were applied. The average methane yields obtained throughout intermittent feeding were lower (0.19 ± 0.03 in R1 and 0.21 ± 0.03 Nm^3^ CH_4_ kg COD_removed_^−1^ in R2) than under continuous feeding (0.31 ± 0.03 in R1 and 0.30 ± 0.02 Nm^3^ CH_4_ kg^−1^ COD_removed_ in R2). Lower production of methane with intermittent substrate feed has been reported in works by Lafita et al. [10] and Torres et al. [21], where treating wastewater with light-oxygenated organic solvents in up-flow granular sludge reactors showed a shift in the biogas production of the anaerobic biomass under intermittent conditions.

**Figure 3:**
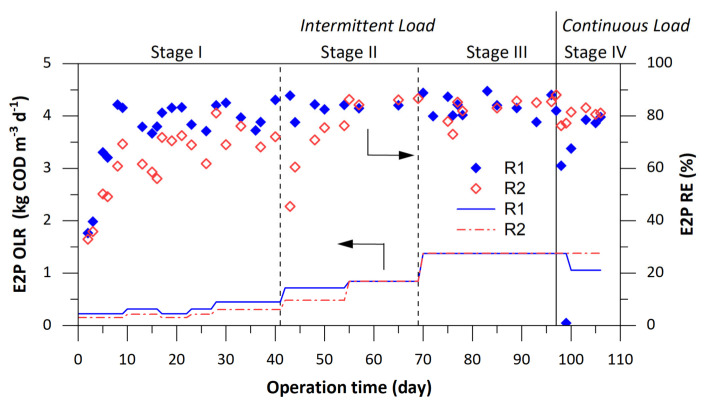
E2P organic load (―) and E2P removal efficiency (♦, **◊**). R1 operated at U_L_ ≤ 0.15 m h^−1^ and R2 operated at U_L_ = 3.0 m h^−1^.

In terms of solvent elimination, removal efficiencies of ethanol and ethyl acetate in both reactors were > 99% since the first day of operation, therefore effluent contained mainly E2P (Figure 3). After some oscillations, E2P removal > 83% was achieved in both reactors under intermittent feeding (Stage III). The change to continuous feeding caused an accumulation of E2P in R1, but at the end of the study E2P removal was > 80%. Intermediate products associated with the anaerobic degradation pathway of E2P are acetone from the ether cleavage and isopropanol from the reversible reduction of acetone in the presence of H_2_ [27]. Both intermediates were detected at low concentrations (acetone < 30 mg COD L^−1^; isopropanol < 20 mg COD L^−1^), thus indicating that the production of the enzyme required for ether cleavage was the limiting step. BMP and SMA were evaluated at the end of the study to assess the methane-producing capability of the granular sludge of both reactors treating each organic solvent (Table 2). As expected, no remarkable differences between the sludges developed in R1 and R2 were observed. BMP values were similar for all substrates (292–341 NmL CH_4_ g COD^−1^). The highest SMA values were obtained with ethanol (574 and 536 NmL CH_4_ g VSS^−1^ d^−1^ in R1 and R2). Similar methanogenic activity was also reported by Enright et al. [28] with values of 594 mL CH4 g VSS^−1^ d^−1^ for the degradation of ethanol with an EGSB granular sludge adapted to psychrophilic treatment of ethanol, acetic acid, methanol, acetone, and propanol. For the tests with ethyl acetate and with the mixture of the three solvents, SMA values were slightly lower than for the ethanol tests (500–520 NmL CH_4_ g VSS^−1^ d^−1^). The lowest biodegradability of E2P was linked to the lowest SMA (3–5 times lower than for ethanol). However, methane production was detected from the beginning, thus a population of microorganisms able to produce the ether cleaving enzymes required for E2P degradation was established [10]. SMA values were better than those obtained by Torres et al. [20] in which anaerobic granulation was done under continuous loading. In the present work, values were 100% greater than for granules obtained in that study without chitosan and 20% greater than in their chitosan-assisted reactors. Therefore, the positive effect of chitosan on granulation and the good characteristics of the granular sludge obtained under intermittent operation are well demonstrated. These results will help to establish a protocol for the start-up of industrial up-flow anaerobic reactors treating light-oxygenated solvents from suspended-growth anaerobic sludge.

### 3.2. Formation of Anaerobic Granules

Table 3 summarizes the evolution of the percentage of granules and mean particle size evaluated from biomass samples taken throughout the study. At the start-up, three weekly doses of chitosan were applied to accelerate the formation of granules. The percentage of granules (particles greater than 300 µm [25]) increased significantly from 5.7% in the inoculum to 62.6% in R1 and 51.9% in R2 after only 20 days of operation along with a 4–5 times increment in the mean particle diameter. Therefore, good granulation was achieved early with intermittent feeding of light-oxygenated solvents in a range of up-flow velocities from a sludge blanket (R1) to an expanded bed (R2). Results corroborate the enhancement of granule formation when chitosan is used in the presence of organic solvents, as other authors have reported. Rapid granulation in the presence of chitosan has been reported by Hudayah et al. [17] in semi-continuously fed reactors with glucose and propionate used as organic substrates. These authors obtained granule percentages of 26.4% after 25 days of operation. After the chitosan dosage on day 21 to reactors R1 and R2, the adhesion of biogas bubbles to the flocs and the partial flotation of the biomass was observed. Chitosan enhances surface hydrophobicity [29], thus promoting cell aggregation, but, at the same time, gas bubbles adhere more effectively to hydrophobic surfaces [30]. Therefore, the periodic addition of chitosan was interrupted, and the flotation disappeared. On day 41 (end of Stage I), a significant decrease in the granule content and size occurred in both reactors even though the process performance was outstanding (RE_COD_ > 90%). Partial disintegration of granules was previously observed in reactors inoculated with granular sludge under a fluctuating supply of wastewater containing similar solvents [10,21]. After the addition of a last dose of chitosan on day 42, the improvement on granule production was demonstrated in the next particle size evaluation (day 61, Stage II). An adequate dosage of chitosan had a positive effect not only on the granulation but also on the restoration of partially disintegrated granules, as previously reported [31]. Further operation under intermittent supply of substrate caused, again, a deterioration of the granules (day 82, Stage III). By contrast, continuous feeding from day 98 rapidly increased the percentage of granules (day 106, up to > 70% in R1 and > 50% in R2) and nearly doubled the average particle diameter. Comparison of granule percentages and mean diameter in both reactors showed slightly more favorable results for R1 operating at lower up-flow velocity. Proper manipulation of up-flow liquid velocity is critical for granule formation [11]. Indeed, selection pressure from hydraulic (and gas) loading rates has been related to washout of light and dispersed sludge while retaining denser particles promote granulation [32]. Therefore, positive effects of the increase of up-flow velocity have been reported for maximum velocities of 0.65 m h^−1^ [33] or 0.5 m h^−1^ [34]. Moreover, velocities > 1 m h^−1^ have been identified as the cause of the disintegration of the granules due to the shear forces inside the reactor [35]. Thus, lower percentage of granules and mean diameter in R2 were derived from the higher shear stress. In any case, successful granulation at 3 m h^−1^ was very promising for the treatment of low-strength solvent wastewater (< 2 g COD L^−1^) and consequently for the implementation of the anaerobic bioscrubber as a VOC control technique.

**Figure 4:**
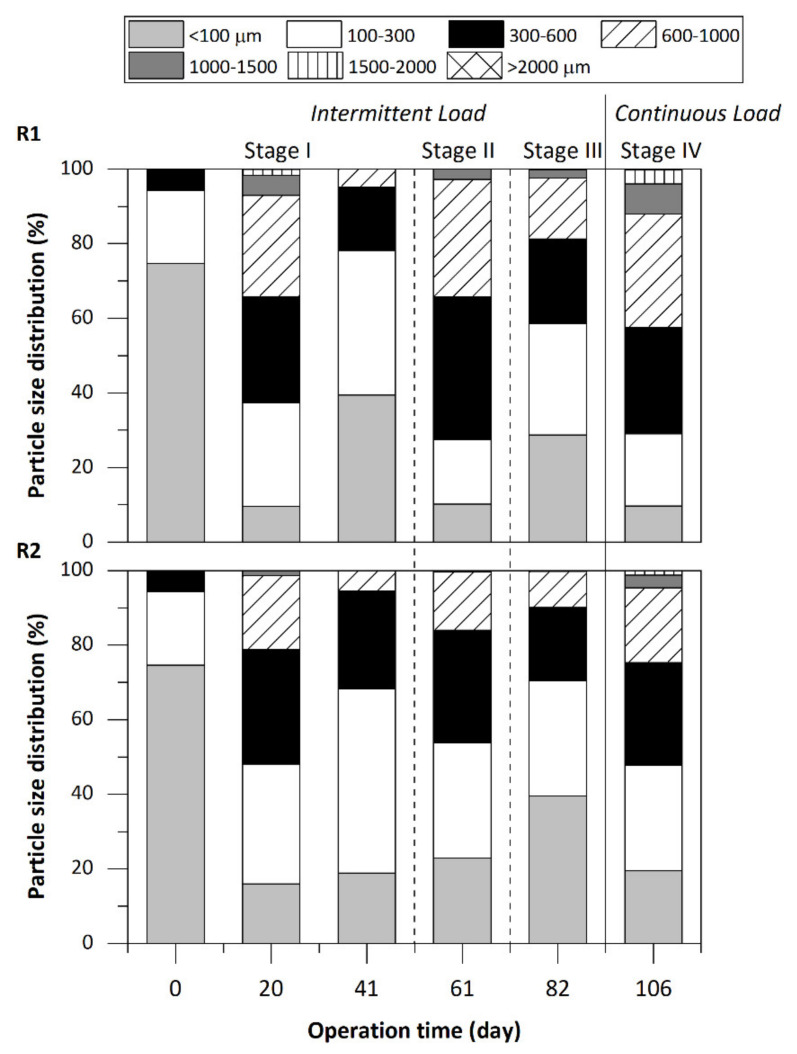
Evolution of the particle size distribution. (**a**) R1, operated at U_L_ ≤ 0.15 m h^−1^, (**b**) R2, operated at U_L_ = 3.0 m h^−1^.

Figure 4 shows the evolution of the particle size distribution in each reactor. The particle size increased significantly in both reactors compared to the inoculum, demonstrating the early formation of aggregates and granules even with sizes > 1000 µm and > 1500 µm. The significant decrease in the percentage of particles with a size < 100 µm and the increase in the percentage of particles with a size of 600–1000 µm are remarkable, reaching percentages of 42.4% in the R1 reactor and 24.6% in reactor R2 on day 106. These results confirm that the addition of chitosan is a very good strategy for the fast start-up of anaerobic sludge bed reactors from cheap and affordable suspended-growth cultures treating solvent-based wastewater intermittently produced. Some fluctuations in particle size distribution were observed during intermittent operation that were mainly related to the periodical dosage of chitosan with more and bigger granules after chitosan addition (day 20 and day 61). Hence, a periodical supplementation with chitosan (every few weeks) could help to improve the robustness of process performance by offsetting the partial deterioration of granules caused by intermittent substrate supply.

**Figure 5:**
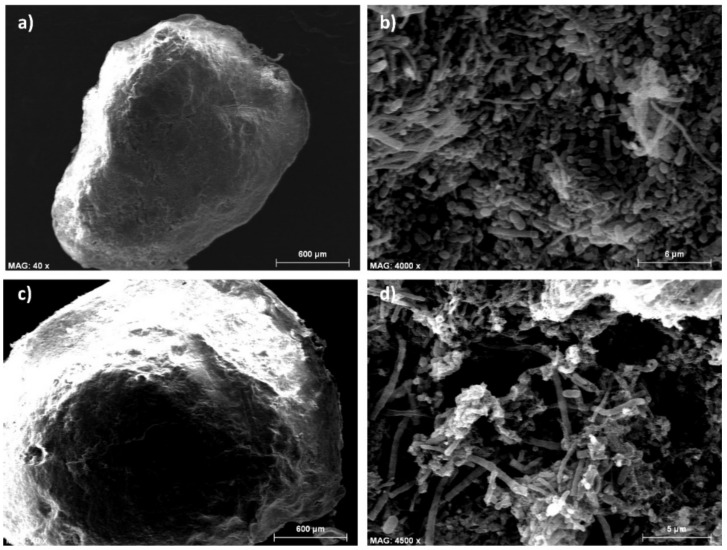
SEM images of morphology of the granules at the end of the study. (**a**,**b**) Granule from the sludge bed of R2, (**c**,**d**) floating granule from the sedimentation zone of R2.

The sedimentation rate of the bed biomass formed and its resistance to shear stress were determined at the end of the study (Table 4). The sedimentation rates of the bed biomass of both reactors, around 20 m h^−1^, were within the typical range describing anaerobic granules of good quality, 15–50 m h^−1^, according to van Lier et al. [36]. Figure 5a,b shows SEM images from a representative granule taken from the bed reaction zone of R2. A rigid structure with ellipsoidal shape (Figure 5a) showing uniform cell density with a mixed population of cocci (spheres), bacilli (rods) and filamentous microorganisms (Figure 5b) corroborated the excellent quality of the granules. Few floating aggregates were accumulated in the sedimentation zone of both reactors at the end of the study. Contrarily, these particles were considerably larger than the granules of the bed (Figure 5c) and the magnification of their surface showed a less uniform cell density with hollow spaces (Figure 5d) possibly due to cellular autolysis derived from organic substrate limitations [36,37]. Integrity coefficient gives a qualitative indication of the shear strength of granules, high strength correlating with low IC values [38]. At low agitation times (IC_2_, IC_3_), granules from the reactor working at the lowest up-flow velocity (R1) showed slightly higher resistance to moderate shear forces. The ascending velocity of 3 m h^−1^ (R2) was slightly detrimental to the structural integrity of the granules, which also correlates to the smaller mean particle diameter of R2. Nevertheless, as agitation time increases to 5 min (IC_5_), the granules’ strength measured in both reactors tended towards very similar values, which indicates that similar erosion of granules would be expected under high biogas production for the two tested up-flow velocities. For example, IC_5_ values ranging from 6.8% to 27% have been reported in UASB reactors fed with sucrose after 240 days of operation using flocculent anaerobic sludge as an inoculum [22]. These authors correlated IC_5_ with optimal OLRs during the start-up, suggesting an OLR range of 2.0–4.5 kg COD m^−3^ d^−1^ to start the reactor when using flocculent sludge. Therefore, the higher values obtained in this study can be associated with the source of wastewater as it is well known that components, such as solvents and dyes, as well as high salinity can adversely impact the granule quality. For example, IC_4_ values for a UASB reactor fed with a high salinity wastewater varied from 46.9% to 50.4% after 148 days of operation [39] when using sieved biomass from a UASB as an inoculum. The chitosan supplementation proposed herein was favorable for the fast formation of granular sludge with moderate IC_5_ (less than 50%) representing enough strength to obtain high COD removal efficiency.

### 3.3. Microbial Community Analysis

**Figure 6:**
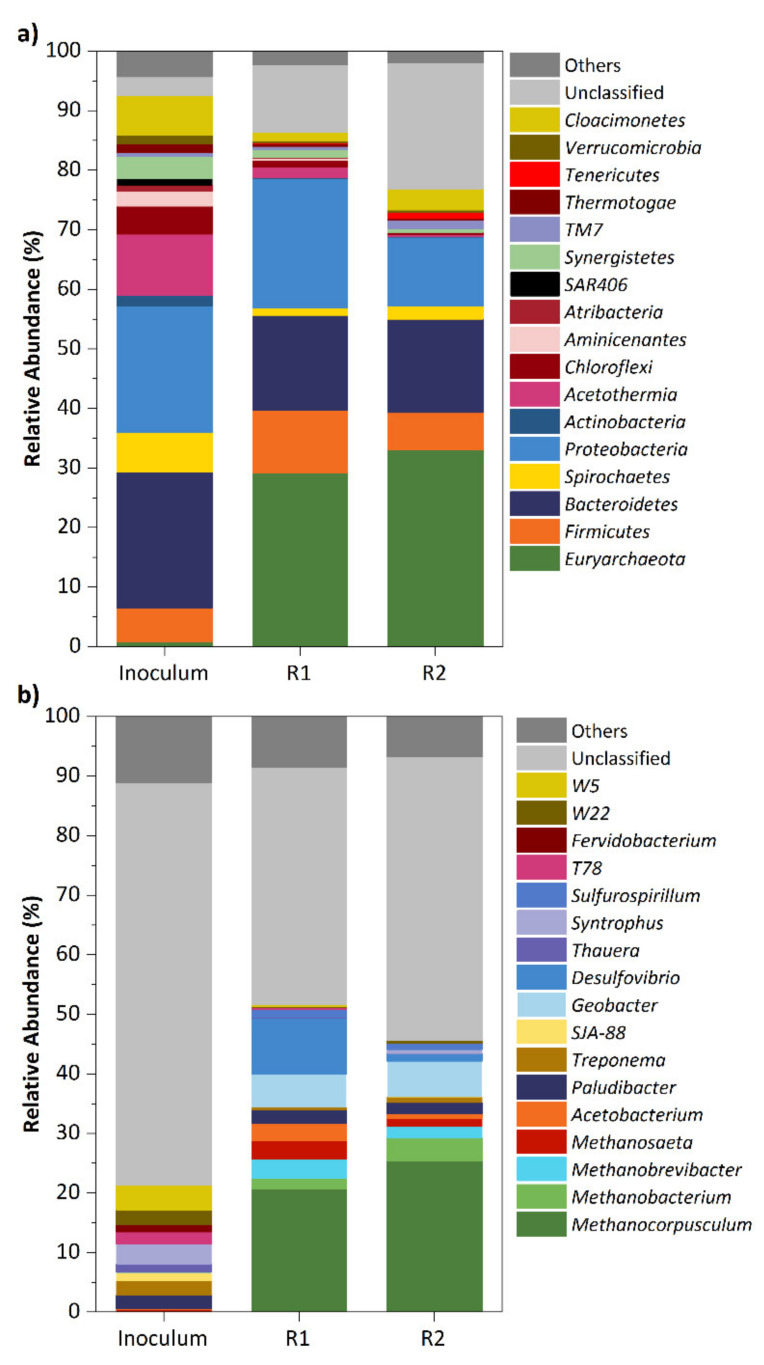
Microbial community structures in the inoculum and in the granular sludge in both reactors at the end of the study: (**a**) at phylum level, (**b**) at genus level.

The sludge samples taken from both reactors at the end of the experiment (day 106) and from the inoculum (day 0) were analyzed via high-throughput sequencing. The microbial community structure at phylum level is shown in Figure 6a (phyla detected in relative abundances higher than 1% in at least one sample). A shift in the microbial communities from the seed sludge to the granules of both reactors was observed after 106 days of operation. *Bacteroidetes*, *Proteobacteria*, *Acetothermia*, *Synergistetes*, *Cloacimonetes*, and *Firmicutes* were the most abundant phyla in the inoculum while at the end of the study both reactors had similar microbial populations being the predominant phyla, *Euryarchaeota*, *Proteobacteria*, *Bacteroidetes*, and *Firmicutes*. The latter phyla have been found dominant in biomass samples from anaerobic reactors treating wastewater with organic substrates [21,40,41,42,43,44,45]. The inoculum’s microbial community exhibited more diversity (Shannon Diversity index of 6.8) than those observed in R1 and R2 with Shannon Diversity indexes of 6.0 and 5.5, respectively, showing the adaptation of the microbial population to the treatment of light-oxygenated solvents under intermittent loading conditions. The small differences between both reactors indicated that differences in mass transfer features of granules due to the different up-flow velocities applied in R1 (sludge blanket) and R2 (expanded bed) had a negligible influence on the microbial structure when compared to the change in substrate from the inoculum. The *Euryarchaeota* phylum, to which methanogenic archaea belongs, became dominant in the granular samples after a remarkable increase in its relative abundance from 0.8% in the inoculum to values of 29.2% and 33.1% in R1 and R2, respectively. *Euryarchaeota* phylum dominance in the granulation of WWTP anaerobic sludge during the treatment of oxygenated solvents has been previously observed [20]. *Bacteroidetes*, which was the most abundant phylum in the inoculum accounting for 22.8%, dropped to 16.0% in R1 and to 15.6% in R2. Species belonging to this phylum are reported to play a role in the hydrolysis and acidogenesis steps in anaerobic degradation [46]. Therefore, its decrease could be explained by the high content of ethanol in the effluent, whose degradation is mainly carried out by syntrophic interactions between acetogenic bacteria and hydrogenotrophic methanogens [47,48]. Other dominant phylum in the inoculum, such as *Proteobacteria* (21.2%), remained in a value of 21.8% in R1 while it decreased its abundance to a value of 11.6% in R2. Contrarily, *Firmicutes* increased the relative abundance over the course of the experiment from 5.7% (day 0) to 10.5% and 6.2% in R1 and R2, respectively. Bacteria of this phylum have been found to be dominant in degradation of organic compounds [49] as well as during the degradation of volatile fatty acids [50].

Figure 6b shows the relative abundance of the dominant genera (detected with abundances higher than 1% at least in one sample). As previously commented, both reactors exhibited a significant change in their communities achieving quite similar populations at the genus level by the end of the experiment. The archaeal communities were composed of hydrogenotrophic methanogens associated with the three genera, *Methanocorpusculum*, *Methanobacterium*, *Methanobrevibacter*, and acetoclastic methanogens of the *Methanosaeta* genus. *Methanocorpusculum* methanogens were dominant in the granules with relative abundances of 20.7% and 25.3% in R1 and R2, respectively, while the remaining methanogens accounted for abundances ranging from 1.3% to 4.0%. The greater abundance of hydrogenotrophic methanogens rather than acetoclastic methanogens suggests that hydrogen methanogenesis was a major route of methane production in the anaerobic treatment of the three light-oxygenated solvents: ethanol, ethyl acetate, and 1-ethoxy-2-propanol. Indeed, previous studies on the treatment of several industrial wastewaters by granular bed reactors have also found dominance of hydrogenotrophic methanogens [20,21,41,43,51]. At the genus level of bacteria, *Paludibacter* was the only one to maintain its relative abundance at values around 2.0% in all samples. This genus that involves fermentative bacteria producing propionate from a wide range of substrates [52,53] has been reported with similar abundances in granules treating municipal wastewater at temperatures around 20 °C [54]. In addition, three abundant genera in the granules were *Acetobacterium*, *Geobacter*, and *Desulfovibrio*, which increased considerably in relative abundance after 106 days of operation from values in the inoculum lower than 0.2%. *Acetobacterium*, which achieved abundances of 3.0% and 0.9% in R1 and R2, respectively, have been previously reported as a candidate to participate in the degradation of ethyl acetate and 1-ethoxy-2-propanol [21]. These microorganisms can degrade some methyl esters to methanol and can perform the enzymatic cleavage of the ether bond of glycol ethers [55,56]. *Geobacter*, with abundance in both reactors around 6.0%, are common microorganisms in anaerobic reactors since they can oxidize ethanol, acetate, formate, or lactate coupled with the reduction of iron or manganese oxides [57]. *Desulfovibrio* genus rose to values of 9.5% and 1.2% in R1 and R2, respectively. The species belonging to this genus can degrade ethanol or lactate in syntrophic association with hydrogenotrophic methanogens [58]. *Geobacter* and *Desulfovibrio* have been found with significant abundance in granules of anaerobic reactors treating wastewater polluted with ethanol [59,60]. The notable growth of species belonging to these three genera (*Acetobacterium*, *Geobacter*, and *Desulfovibrio*) corroborates their important role in the treatment of light-oxygenated solvents.

## 4. Conclusions

This is the first study on granulation under intermittent wastewater in chitosan-assisted up-flow anaerobic reactors inoculated with flocculent sludge. This research confirms that the addition of chitosan during the start-up is a very effective strategy for a fast formation of compact granules (in less than 3 weeks), achieving high COD removal efficiencies while treating a mixture of ethanol, ethyl acetate, and 1-ethoxy-2propanol, solvents typical in the printing sector. The addition of chitosan rapidly improved the solid retention independently of the fluid hydrodynamics established, demonstrating that this strategy can be applied to both UASB and EGSB reactors operating at sub-mesophilic temperatures. Microbial community analysis showed a strong shift in the structure diversity from the inoculum. *Methanocorpusculum* hydrogenotrophic methanogens became dominant in the granules along with *Acetobacterium*, *Geobacter*, and *Desulfovibrio* bacteria, thus demonstrating the role of syntrophic association between bacteria and archaea in the degradation of these solvents. Intermittent substrate supply was shown detrimental for the granule maturation while periodic chitosan addition (every few weeks) could enhance the robustness of the process, thus keeping high removal efficiencies when treating OLR up to 13 kg COD m^−3^ d^−1^ of a mixture of light-oxygenated solvents.

## Figures and Tables

**Table 1:** Operational schedule of the anaerobic sludge bed reactors.

	Stage I		Stage II		Stage III		Stage IV	
Operational day	0–41		42–69		70–97		98–106	
Reactor	R1	R2	R1	R2	R1	R2	R1	R2
Operational mode	Intermittent load 16 h d^−1 a^						Continuous load	
OLR (kg COD m^−3^ d^−1^) ^a^	2.1–4.3	1.4–2.9	6.8–8.0	4.6–8.0	13.0	13.0	10.0	13.0
Influent COD (g L^−1^)	4.3	2.9–2.6	4.3–5.1	3.6–5.1	8.3	8.3	6.4	8.3
Up-flow velocity, U_L_ (m h^−1^)	0.05–0.10	3.00	0.12–0.15	3.00	0.15	3.00	0.15	3.00

^a^—instantaneous organic loading rate applied during 16 h per day.

**Table 2:** Biochemical methane potential and specific methanogenic activity of the granular sludge of each reactor at the end of the study.

	**BMP (NmL CH_4_ g COD^−1^)**			
Reactor	Ethanol	Ethyl Acetate	E2P ^a^	Mixture
R1	299 ± 5	292 ± 5	341 ± 9	300 ± 5
R2	303 ± 4	308 ± 10	321 ± 5	314 ± 13
	**SMA (NmL CH_4_ g VSS^−1^ d^−1^)**			
Reactor	Ethanol	Ethyl Acetate	E2P ^a^	Mixture
R1	574 ± 20	520 ± 13	110 ± 1	510 ± 6
R2	536 ± 6	518 ± 19	142 ± 11	500 ± 6

^a^—E2P, 1-ethoxy-2-propanol.

**Table 3:** Evolution of the granule content and the particle size (quantified in terms of De Brouckere mean diameter) of the sludge samples in each reactor.

		Granules (%)		Mean Diameter (µm)	
	Day	R1	R2	R1	R2
Stage I	0	5.7	5.7	85	85
	20	62.6	51.9	475	354
	41	21.8	31.6	187	238
Stage II	61	72.4	46.3	465	307
Stage III	82	41.4	29.5	312	224
Stage IV	106	71.1	52.2	562	386

**Table 4:** Characteristics of developed granules at the end of the study.

			Granular Strength		
Reactor	Mean Diameter (µm)	Settling Velocity (m h^−1^)	IC_2_ (%) ^a^	IC_3_ (%) ^a^	IC_5_ (%) ^a^
R1	562	19.9	13	18	38
R2	386	22.4	24	33	43

^a^—IC_t_, integrity coefficient percentage ratio evaluated after t minutes of agitation.
